# Phage vB_YenS_P400, a Novel Virulent Siphovirus of *Yersinia enterocolitica* Isolated from Deer

**DOI:** 10.3390/microorganisms10081674

**Published:** 2022-08-19

**Authors:** Jens A. Hammerl, Andrea Barac, Claudia Jäckel, Julius Fuhrmann, Ashish Gadicherla, Stefan Hertwig

**Affiliations:** Department of Biological Safety, German Federal Institute for Risk Assessment, Max-Dohrn Str. 8-10, D-10589 Berlin, Germany

**Keywords:** *Yersinia enterocolitica*, phage, genome, siphovirus, virulent, host range

## Abstract

Phage vB_YenS_P400 isolated from deer, is a virulent siphovirus of *Y. enterocolitica*, whose circularly permutated genome (46,585 bp) is not substantially related to any other phage deposited in public nucleotide databases. vB_YenS_P400 showed a very narrow host range and exclusively lysed two *Y. enterocolitica* B4/O:3 strains. Moreover, lytic activity by this phage was only discernible at room temperature. Together with the finding that vB_YenS_P400 revealed a long latent period (90 to 100 min) and low burst size (five to ten), it is not suitable for applications but provides insight into the diversity of *Yersinia* phages.

## 1. Introduction

Yersiniosis is an important foodborne gastrointestinal disease that is mainly caused by *Yersinia* (*Y*.) *enterocolitica* [1]. This species is well adapted to pigs, which represent a natural reservoir for the bacteria [2,3]. Thus, it is not surprising that most human infections are caused by the consumption of raw or insufficiently cooked pork [4]. However, other food products like vegetables (i.e., carrots, mixed salad) that can be contaminated with the bacteria have also been reported as possible sources of infection [5,6,7]. Contamination may occur by, e.g., game animals, whose tonsils and gut are quite frequently populated by yersiniae [8,9]. The species *Y. enterocolitica* is comprised of six biotypes (B: B1A, B1B, B2, B3, B4, and B5) and more than 70 serotypes (O) [10]. In Europe, the most clinically important bio/serotypes are B4/O:3, B2/O:9 and B2/O:5,27, whereas biotype B1A which frequently occurs in the environment, animals, and also in food is considered to be nonpathogenic, even though there are some reports on the isolation of this biotype from sick persons [11,12]. To combat pathogenic yersiniae along the food chain, a number of virulent phages have been described, almost all of which are podoviruses and myoviruses. Most podoviruses infecting *Y. enterocolitica* are T7-like phages having a restricted host range as they mainly lyse strains of serotype O:3 [13,14,15]. An exemption is the T7-like phage vB_YenP_Rambo that infected 50 out of 62 pathogenic *Y. enterocolitica* strains belonging to several bio/serotypes at 28 °C [16]. At 37 °C, the phage even lysed more pathogenic strains and additionally six out of ten B1A strains. In general, *Y. enterocolitica* myoviruses exhibit a broader host range than podoviruses since they are additionally able to lyse strains of O:9 and O:5,27, though some of these phages were active only at and below 25 °C [17]. The widest host range of all *Yersinia* phages described thus far revealed the myovirus PY100 and its close relative vB_YenM_P281 isolated from manure and game, respectively [16,18]. These two phages not only infect various bio-/serotypes of *Y. enterocolitica* but also strains of *Y. pseudotuberculosis* if the medium is supplemented with calcium and magnesium cations [16]. Contrary to podoviruses and myoviruses, to date, only one virulent *Yersinia* siphovirus (phiR2-01) has been reported [19]. Phage phiR2-01 has a genome of 122,696 bp with long terminal repeats. It is similar to T5 and able to lyse a broad range of *Y. enterocolitica* strains of different serotypes at room temperature. The host receptor of phiR2-01 is the outer membrane vitamin B12 receptor BtuB.

In this work, we have characterized the siphovirus vB_YenS_P400. Unlike phiR2-01, phage vB_YenS_P400 is a novel phage, whose genome does not show any substantial nucleotide similarity to previously published phage genomes.

## 2. Materials and Methods

### 2.1. Bacterial Strains and Culture Conditions

The strains used here originate from the culture collection of the Consiliary Laboratory for *Yersinia* (KL *Yersinia*) at the German Federal Institute for Risk Assessment (BfR), Berlin, Germany. Bacteria were cultivated in/on lysogeny broth (LB)-based media at RT, 28 °C or 37 °C, as specified [20].

### 2.2. Isolation, Propagation, and Purification of Phages

vB_YenS_P400 was isolated from a fecal sample of a deer hunted in northeast Germany in the season 2019/2020. Five milliliter SM buffer was added to 1 g feces and incubated on a stirrer at 4 °C overnight [21]. Sterilization of the sample was achieved by centrifugation for 20 min at 8000 rpm and 10 °C followed by a successive filtration through 0.45 and 0.22 μm pore-size filters (VWR International, Darmstadt, Germany). The lytic activity of the phage was determined by spot assays. For this, 10 μL of serial dilutions of the sample were applied onto the lawn of *Y. enterocolitica* indicator strains belonging to various bio-/serotypes. After incubation overnight at RT, 28 °C, and 37 °C, agar plates were inspected for plaque formation. vB_YenS_P400 plaques were subjected to a three-fold plaque purification procedure. High-titer lysates of the phage were obtained by infecting 200 mL cultures of the indicator strain (OD_588_ = 0.5) *Y. enterocolitica* B4/O:3 strain DSM 9676 or by preparing 10–20 agar plates with confluent lysis of the host bacteria. The soft agar was harvested by scraping and resuspended in SM buffer for several hours. Thereafter, the lysates were centrifuged for 20 min at 10,000× *g* to remove agar and debris and then filtered (see above). For purification, vB_YenS_P400 particles were concentrated by ultracentrifugation and applied onto a CsCl step gradient as previously described [22].

### 2.3. Host Range Determination

The host range of vB_YenS_P400 was determined by spot activity assays. For this, 100–200 μL of each indicator strain were mixed with 6 mL prewarmed NZCYM (VWR International, Darmstadt, Germany) soft agar (0.6%) and poured onto an LB agar plate. Ten microliters of a serial dilution series of the vB_YenS_P400 lysate were spotted onto the overlay agar and evaluated after overnight incubation at RT, 28 °C, or 37 °C [16]. The specificity of vB_YenS_P400 was tested on 50 *Y. enterocolitica* strains, 30 strains belonging to other *Yersinia* species, and non-*Yersinia* strains representing various other genera of *Enterobacterales* (e.g., *Escherichia* spp., n = 10; *Klebsiella* spp., n = 10; *Salmonella*, n = 10 and other species, *Proteus mirabilis*, n = 2; *Enterobacter cloacae*, n = 2; *Morganella morganii*, (n = 2 and *Citrobacter freundii*, n = 2). Further information on the tested isolates is given by Hammerl et al. (2022) [23].

### 2.4. Lysogenization Experiment

100 μL of a high-titer lysate (10^9^ pfu/mL) of the phage were spotted onto a lawn of strain DSM 9676 (see 2.3) resulting in a very clear zone of lysis. Using a sterile toothpick, some material was removed from the lysis zone, resuspended in 100 μL SM buffer, and plated onto an LB agar plate. After incubation overnight at 28 °C, 20 colonies were isolated and streaked twice onto LB agar to get rid of phages, which had possibly bound to the cells. The isolates were then used for both, mitomycin C treatment to induce prophages and PCR applying primers deduced from the vB_YenS_P400 genome.

### 2.5. Transmission Electron Microscopy (TEM)

CsCl-purified phages were investigated by TEM using the negative staining procedure with uranyl acetate (VWR International, Darmstadt, Germany) as previously described [22]. Specimens were examined by TEM using a JEM-1010 (JEOL, Tokyo, Japan) at an 80 kV acceleration voltage [24].

### 2.6. Phage DNA Preparation, Sequencing, and Genome Analysis

The phage genome was sequenced by short-read, paired-end whole-genome sequencing. Phage DNA was prepared from proteinase K/SDS treated vB_YenS_P400 virons, as previously described. A phage DNA sequencing library was prepared with the DNA Flex Library Preparation kit (Illumina, San Diego, CA, USA) and sequenced on an Illumina NextSeq500 device, as previously described [16]. Quality assessment of raw sequencing reads was conducted using the Aquamis pipeline [25]. The de novo assembling and coding sequence prediction was performed using the spades algorithm of the Pathosystems Resource Integration Center (PATRIC) database (version 3.6.20). In general, sequence comparison was carried out using the blast-suite (https://blast.ncbi.nlm.nih.gov/Blast.cgi) of the National Center for Biotechnology Information (NCBI). For the prediction of transcription terminators, the ARNold software (http://rssf.i2bc.paris-saclay.fr/toolbox/arnold/) was used. All software tools mentioned here were used with default settings. Basic sequence analysis was conducted using DS Gene (version 2.5; Accelrys Inc., San Diego, CA, USA) [16].

### 2.7. Determination of the Page DNA End Structure

Phage DNA was analyzed by enzymatic digestions for the determination of the end structure. To examine the genome for cohesive ends, EcoRV restriction patterns of untreated, heated (80 °C, 10 min, followed by short incubation on ice), and T4-ligated phage DNA were compared. Bal31 exonuclease (Thermo Fisher Scientific, Schwerte, Germany) digestion was performed to check whether the phage DNA is circularly permuted. The exonuclease treatment was carried out for 7.5, 15, 30, 60, and 120 min at 30 °C. Immediately after digestion, Bal31 was inactivated by incubation for 10 min at 60 °C. After ethanol precipitation, the DNA was cleaved with EcoRV for 20 min at 37 °C. Restriction patterns of the digested DNAs were analyzed by gel electrophoresis on 0.8% agarose gels [26].

### 2.8. Nucleotide Sequence Accession

The genome of the *Y. enterocolitica* phage vB_YenS_P400 was deposited in GenBank under the accession number OK042081.

## 3. Results and Discussion

### 3.1. vB_YenS_P400 Is a Siphovirus Originating from Wildlife

**Figure 1:**
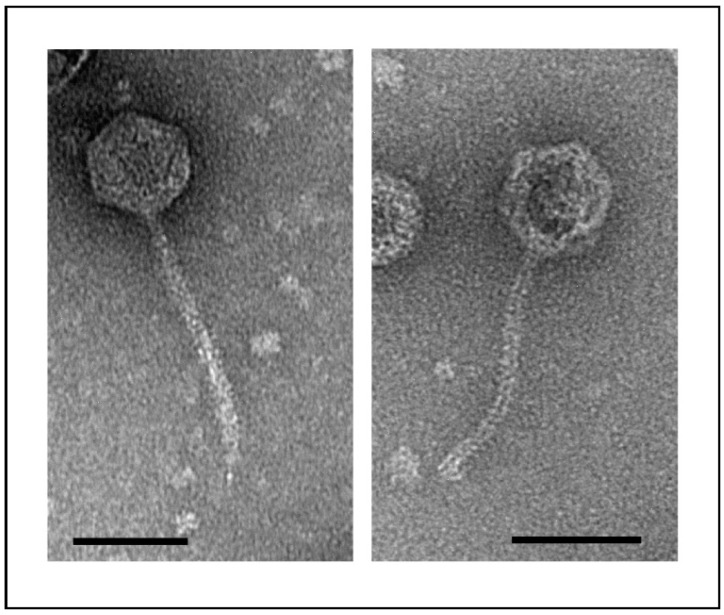
Electron micrograph of phage vB_YenS_P400. The bar represents 50 nm.

Phage vB_YenS_P400 was isolated in November 2019 from the feces of a female deer hunted in Brandenburg, Germany. Previous studies indicated that game animals may be a valuable source of new *Yersinia* phages [16]. However, contrary to several myoviruses and podoviruses, which we could isolate from wild animals, electron microscopy revealed that phage vB_YenS_P400 has a long, non-contractile tail and is thus a siphovirus (Figure 1). Its head diameter is 58.6 ± 6 nm, while its tail length is 117.4 ± 21 nm. This phage is to date the only *Yersinia* siphovirus originating from game.

### 3.2. vB_YenS_P400 Exhibited a Narrow Host Range

**Figure 2:**
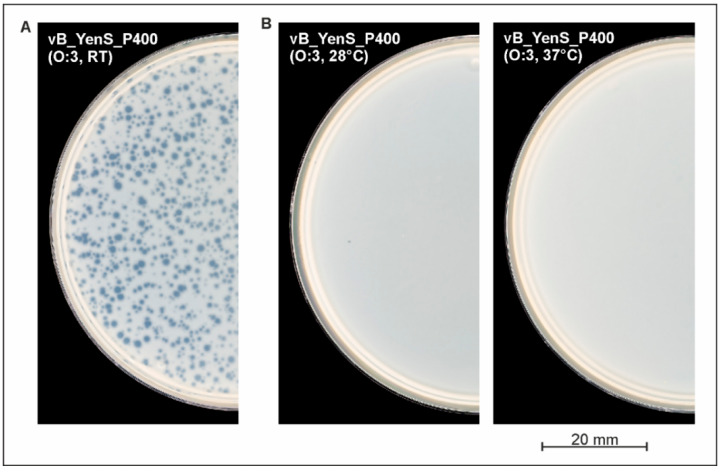
Plaque formation by the phage on *Y. enterocolitica* B4/O:3 strain DSM 9676 only occurred at room temperature (**A**), while incubation at 28 °C and 37 °C did not yield in plaques (**B**).

To determine the host range of vB_YenS_P400, a total of 120 strains (50 *Y. enterocolitica*, 30 strains belonging to other *Yersinia* species, and 40 strains of other *Enterobacterales* genera were examined. The phage revealed an extremely narrow host range and exclusively lysed two B4/O:3 strains, one of which is the reference strain DSM 9676. Moreover, it showed lytic activity only at room temperature, whereas at 28 °C and 37 °C, no lysis was observed (Figure 2). We also tested LB soft agar and the influence of CaCl_2_ and MgSO_4_ cations (20 mM each), which may be important for plaque formation [16] but could not detect a change in the lytic properties of the phage.

In this regard, vB_YenS_P400 resembles temperate *Y. enterocolitica* phages [23], and the virulent *Yersinia* myoviruses phiR1-RT and vB_YenM_TG1 which, however, have a much broader host range [17]. These two phages use OmpF as a receptor that is maximally expressed at 4 °C and strongly repressed at 37 °C. The question arises about how vB_YenS_P400 can propagate in the gut of a deer and why it exhibits such a high host specificity. To elucidate whether vB_YenS_P400 is able to bind to susceptible strains at elevated temperatures and to an insusceptible strain, we determined the adsorption of the phage to strain DSM 9676 at 37 °C and to another O:3 strain, which is not lysed by the phage. For this study, various dilutions of a lysate were used to obtain meaningful results. Adsorption was monitored for up to 120 min. In none of the experiments was a significant difference in adsorption of the samples detected even after 90 min of incubation, suggesting that binding of vB_YenS_P400 to the host is slow, but neither restricted at 37 °C nor by use of a non-susceptible strain (data not shown). After 90 to 100 min of infection, the number of plaques increased strongly only with strain DSM 9676 at room temperature indicating a long latent period of the phage and a burst size of five to ten (Table 1). This result shows that the phage is able to lyse the susceptible strain in broth at room temperature but that it is still unclear which factor determines the insensitivity of strain DSM 9676 at higher temperatures and to other strains. Since only two out of ten tested B4/O:3 strains were lysed, it is rather unlikely that the O-antigen is the receptor for the phage. However, it certainly cannot be ruled out that in the gut bacteria other than *Yersinia*, which were not tested in this study, may be infected by vB_YenS_P400. Some *Y. pseudotuberculosis* phages isolated from fecal samples of birds and mammals were able to lyse various genera of *Enterobacteriaceae* [24].

### 3.3. vB_YenS_P400 Is a Novel Virulent Siphovirus

**Figure 3:**
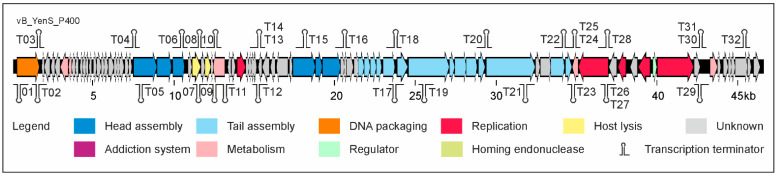
Gene map of vB_YenS_P400.

To determine the relationship to other phages, the whole-genome sequence of vB_YenS_P400 was analyzed. The genome (46,585 bp) contains 84 putative Open Reading Frames (ORFs), of which 60 are located on the positive and 24 on the negative strand. The phage does not show a close relationship to any other phage at the nucleotide level. Though, out of 84 putative gps, the function of 38 proteins could be predicted. They are 39% to 61% identical to proteins of other phages, most of them siphoviruses infecting *Salmonella*. At least 19 predicted vB_YenS_P400 gps may be involved in virion assembly (Figure 3). It is striking that several ORFs probably encoding head proteins are separated by other ORFs (e.g., for lysis proteins) and by numerous predicted transcription terminators. Besides ORFs for structural proteins, a number of ORFs probably encoding proteins important for replication, recombination, metabolism, and packaging of the phage DNA as well as for cell lysis were identified. It is noteworthy that this phage encodes a type III restriction endonuclease (ORF70), which may act in concert with a methyltransferase domain-containing protein (ORF07). The phage also contains a gene (ORF76) possibly belonging to a toxin–antitoxin system. The fact that no integrase gene or a genetic switch were identified on the genome indicates a strictly lytic lifestyle. However, since vB_YenS_P400 is a novel phage, we tried to lysogenize the indicator strain DSM 9676 to clarify whether it is really virulent (see Materials and Methods). Twenty isolates that survived an infection showed resistance to vB_YenS_P400. Though, neither by mitomycin C treatment nor by PCR, the phage or its DNA could be detected in the isolates indicating that it is virulent (data not shown).

### 3.4. The vB_YenS_P400 Genome Is Circularly Permutated

**Figure 4:**
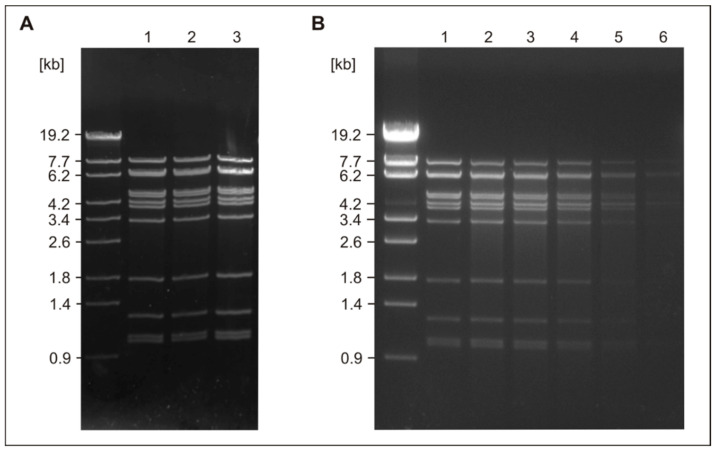
Determination of the DNA end structure of the phage genome. (**A**) Examination of cohesive ends; lane 1, lambda StyI marker, lane 2, EcoRV restriction pattern of untreated phage DNA, lane 2, EcoRV digest, heated at 80 °C and then chilled on ice, lane 3, EcoRV digest of T4-ligated phage DNA. (**B**) EcoRV restriction patterns of phage DNA previously treated by Bal31 for 0 (lane 1), 7.5 (lane 2), 15 (lane 3), 30 (lane 4), 60 (lane 5), and 120 min (lane 6).

Sequence analysis of the phage genome revealed that its terminase belongs to the family of PBSX terminases. As only scarce information exists on this type of terminases and the genome ends, that they generate, we determined the structure of the vB_YenS_P400 genome ends experimentally. For this study, EcoRV was used which cleaves the phage genome at 12 positions. Figure 4A (lane 1) presents the EcoRV restriction pattern of untreated DNA. Fragments with the same intensity were obtained suggesting that the phage does not possess cohesive ends. To confirm this assumption the phage DNA was treated with T4 ligase before digestion with EcoRV. In addition, digested DNA was heated for 10 min at 80 °C and then immediately chilled on ice. Both preparations yielded the same restriction patterns as the untreated DNA confirming the absence of cohesive ends (Figure 4A, lanes 2 and 3). We then used Bal31 digestions to test the phage genome for terminal redundancy. Figure 4B shows a constant decrease of all restriction fragments after 30, 60, and 120 min of incubation with Bal31 demonstrating that the vB_YenS_P400 phage genome is circularly permuted (Figure 4B, lanes 4, 5, and 6).

## 4. Conclusions

The first siphovirus infecting *Y. enterocolitica* was PY54, a temperate phage, which, at the lysogenic state, replicates as a linear plasmid with covalently closed ends [27,28,29]. The first virulent *Yersinia* siphovirus mentioned in the scientific literature is phage R2-01, whose genome organization is similar to that of Enterobacteria phage T5 [19]. Hence, vB_YenS_P400 is the second and novel virulent siphovirus with a genome of 45.6 kb that is not related to any other phage. The closest relatives are some *Salmonella* phages, to whose gene products a number of vB_YenS_P400 proteins are related. One striking feature of the phage is its narrow host range and its inability to lyse *Y. enterocolitica* strains at 28 °C and 37 °C. Thus, vB_YenS_P400 is certainly not a promising candidate for applications along the food chain. However, its strongly restricted host range, which is obviously not caused by the absence of a suitable receptor, suggests an unusual mode of action leading to the insensitivity of most strains. We will try to identify this mechanism by testing transposon mutants of those strains to learn more about phage resistance, which plays an important role in the application of phages.

## Figures and Tables

**Table 1:** Determination of the vB_YenS_P400* burst size and latent period on *Y. enterocolitica* B4/O:3 strain DSM 9676.

Time Point (min)	0	60	65	70	75	80	85	90	95	100	105	110	115	120	180
Test A (pfu) *	16	13	12	14	13	18	21	49	96	155	199	238	264	368	900
Test B (pfu) *	13	17	15	11	23	21	33	58	104	149	205	228	242	340	900
Mean (pfu)	14.5	15	13.5	12.5	18	19.5	27	53.5	100	152	202	233	253	354	900

* A vB_YenS_P400 titer of 1–2 × 10^2^ pfu/mL was used for the determination of the burst size. Intervals of 5 min were used for plaque testing (100 μL). The time period representing an increase in the plaque number is indicated in grey.

## Data Availability

The nucleotide sequence of the phage genome is available at Genbank under accession numbers as indicated above.
